# The future of free flap monitoring by laser continuous doppler flowmetry: A prospective assessment in consecutive 71 patients

**DOI:** 10.1016/j.jpra.2024.11.004

**Published:** 2024-11-17

**Authors:** Hiroki Kodama, Katsuhiro Ishida, Haruyuki Hirayama, Doruk Orgun, Kazuho Kawashima, Dariush Nikkhah, James May, Panicos A Kyriacou, Takeshi Miyawaki

**Affiliations:** a Department of Plastic and Reconstructive Surgery, The Jikei University School of Medicine, Tokyo, Japan; b Research Centre for Biomedical Engineering, City, University of London, London, United Kingdom; c Division of Surgery and Interventional Science, University College London, London, United Kingdom; d Department of Plastic and Reconstructive Surgery, Royal Free London NHS Foundation Trust, London, United Kingdom

**Keywords:** Laser Doppler flowmetry, Free flap, Perfusion monitoring, Head and neck reconstruction, Microcirculation, Non-invasive monitoring, Early detection, Vascular complications

## Abstract

**Objective:**

This study evaluated the effectiveness of laser Doppler flowmetry (LDF) in detecting perfusion disturbances during microvascular free tissue transfer.

**Methods:**

Conducted at a single centre from December 2020 to September 2022, this prospective study involved 71 patients mainly undergoing head and neck free flap reconstructions, using the Pocket LDF™ for continuous perfusion monitoring.

**Results:**

Out of the 71 cases, data from 69 cases were analysed after excluding those with significant noise or sensor detachment. Blood flow disturbances were observed in 9 cases (13.0 %), with 5 of these cases with a history of surgery or radiation in the same area. There were 5 cases of ischaemia, 4 of which occurred during monitoring. There were 4 cases of venous congestion, with 1 occurring during monitoring. Re-operation was necessary in 8 cases (11.6 %), involving flap replacements, vascular re-anastomoses and hematoma evacuation. Complete flap necrosis occurred in 5 cases (7.2 %) and partial necrosis occurred in 3 cases (4.3 %). The LDF device demonstrated the ability to identify perfusion issues hours before the clinical symptoms manifested, suggesting its potential for early intervention. However, challenges included maintaining continuous monitoring immediately post-surgery and during patient transfers.

**Conclusion:**

LDF is a valuable, non-invasive tool for early detection of perfusion disturbances in free flap procedures. It provides continuous, real-time feedback on microcirculation, facilitating timely interventions. Despite its benefits, enhancements in sensor adhesion and wireless technology are needed to improve monitoring reliability. Further studies are recommended to refine LDF usage and validate its efficacy in various clinical settings.

## Introduction

Free flaps are units of tissue that can be moved from one location (donor site) to another (recipient site) while retaining their own blood supply. Free flap reconstruction is an important method for repairing large and complex defects, owing to its superior functional and aesthetic outcomes.^1^ Free tissue transplantation became widely used to correct abnormalities in different body parts, including the head and neck,^1^ breast^2^ and extremities.^3^ Despite the significant advantages of microvascular tissue transplantation, blood flow deficits occur approximately in 3-5 % of the cases.^1^^,^^4^ Vascular thrombosis is a significant risk factor for these transplants, typically manifesting as thrombosis during the first 48 h following surgery.^5^ A severe issue is the delayed diagnosis of vascular thrombosis, which increases the possibility of free flap salvage failure.^6^ Such vascular compromise may lead to free flap loss which requires immediate re-operation, additional surgeries and delayed recovery. Furthermore, flap failures may impact daily activities and the quality of life of the patients and operator.^7^ Past studies report that prompt salvage surgeries after early identification within the first 24 h of the vascular compromise can successfully save 70 % to 80 % of flaps.^4^^,^^8^ For early identification, the gold standard for evaluating a free flap is a conventional clinical examination, which includes measurement of skin tone, capillary refill, turgor, temperature and pin prick.^9^ However, as these observations are made manually and frequently, they are labour intensive.^10^ These characteristics are primarily monitored qualitatively and subjectively, depending on the expertise of the doctor or nurses.^10^ Moreover, as different people monitor the flap, these observations may be inconsistent and susceptible to missing subtle changes; therefore, these observations may also be inconsistent.^11^^,^^12^

Creech and Miller laid out what they thought was the best flap perfusion monitoring system in 1975.^13^ This approach should be accurate, dependable, applicable to all free flaps, simple for healthcare professionals to use, safe for patients and the free flap and allow for early identification. Swartz et al. advocated that the ideal method of monitoring should provide continuous recordings of anastomosis patency and blood flow, distinguish arterial and venous abnormalities and be applicable to buried and cutaneous free flaps.^14^ All modern flap monitoring studies attempt to meet these criteria. Currently, the market offers numerous monitoring devices. For pinpoint measurements, devices such as near-infrared spectroscopy (NIRS),^15^ laser Doppler flowmetry (LDF),^16^ implantable Doppler,^17^ flow couplers^18^ and microdialysis^19^ are available. For imaging-based measurements, indocyanine green angiography^20^ and thermography^21^ are used. Each of these technologies has its strengths and limitations, and there is no universal consensus on their use.^22^ LDF uses the Doppler effect, applying it to laser light instead of ultrasound to detect the variations in wavelength as the photons interact with moving red blood cells.^6^ LDF is particularly effective in monitoring free tissue transfers by providing continuous, non-invasive and immediate feedback on the microcirculation in the free flaps. We have monitored free flaps using LDF and obtained several important findings, which we report herein.

## Methods

**Figure. 1:**
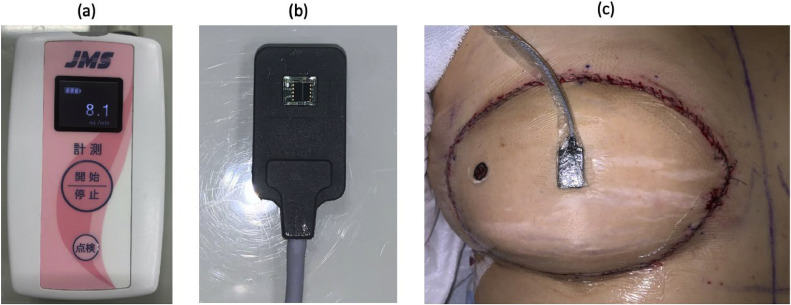
(a) Monitoring unit. Blood flow is shown in ml/min. (b) Sensor unit. The sensor has dimensions of approximately 2 × 1 cm with a laser aperture, which emits a laser of 850 nm wavelength and detects the reflected light. (3) The sensor is placed in the center of the flap within 1 h after surgery.

The present study was approved by The Jikei University School of Medicine Ethics Committee (Application Permit Number; 32-347(10434)). A single-institution, prospective study of patients undergoing free flap reconstruction from December 2020 to September 2022 was performed at The Jikei University Hospital. Data were collected by 4 head and neck reconstructive surgeons (KI, HK, HH and DO). All patients who were expected to undergo free flap reconstruction surgery were invited to participate in the study. As the LDF sensor requires direct contact with the skin, buried flaps were not included. Flaps were continuously monitored using the Pocket LDF™ main unit and sensor probe (Figure 1) manufactured by JMS, Inc.^23^ Data from the sensor were automatically collected by the system every second.

The sensor was affixed to the centre of the flap using a biocompatible double-sided tape, supplied by the manufacturer, within 1 h after surgery. Measurements were performed continuously and were only interrupted occasionally for patient transportation, due to probe dislocation or to correct signal interferences due to blood or wound exudate collecting underneath the measuring probe. It was challenging to maintain continuous measurement immediately post-surgery owing to the difficulty in keeping a personal computer (PC), which receives the recordings via Bluetooth from the main unit, within a few metres during the transition of the patient's bed to the intensive care unit (ICU). Thus, consistent data recording could not be commenced immediately after the operation. Similarly, maintaining proximity to the recording PC was challenging when transferring the patient from the ICU to the ward the following morning. Consequently, recording was completed when the patient was moved to the ward. On an average, the length of time measured was approximately 10 h, although the duration varied based on patient situations. As there were no previous studies on flap monitoring using the Pocket LDF™, LDF was introduced experimentally as a mere research tool. Thus, blood flow measurements were not considered during the flap assessment.

While undergoing LDF monitoring, patients were also monitored via the routine post-flap protocol performed at our institution. In our facility, the nursing staff performed checks every hour and clinical assessments were conducted every 3 h. If any abnormalities were suspected, the anastomosis site was examined using a handheld Doppler probe or an ultrasound probe. Patient data were excluded from the analysis in case of incomplete or corrupted data, very short or interrupted data or data from only one sensor.

## Results

In this study, a cohort of 71 patients was included. Two cases were excluded, as 1 patient had involuntary movements of the tongue that caused significant noise, and another patient experienced peeling off of the intra-oral sensor post-operatively that could not be reattached. Eventually, 69 cases were included in the analysis.

**Figure. 2:**
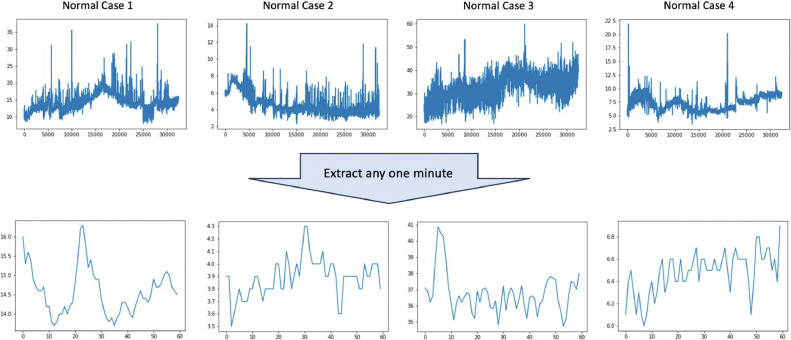
In all graphs, the vertical axis is in ml/min and horizontal axis is in s. The waveforms for 4 representative flaps that survived are shown above for the whole period and below for any 1 min.

The demographic and clinical characteristics of the participants are detailed in Table 1. The total measurement time was 41,588 (3,804–86,401) s and mean blood flow was 8.96 ml/min (1.50-34.9 ml/min) (Table 2). The blood perfusion trajectories for 5 arbitrarily selected typical cases are illustrated in Figure 2, highlighting the total measurement duration and specific one-minute intervals.

**Table 1:** Demographics (n=69).

Characteristics	Value (%)
Age, years	
Median	66
Range	25-90
Gender	
Male	50 (72.5)
Female BMI, kg/m^2^ Median Range Radiation history Yes	19 (27.5) 22.0 15.5-33.8 17
No	52
Primary site Mandibular Maxilla Tongue External auricular Breast Face Others Type of free flap RAMC Fibula DIEP ALT Dorsalis major Forearm Others Recipient Artery Superior thyroid artery Temporal artery Facial artery Internal mammary artery Transcervical artery Others Recipient Vein Internal jugular vein External jugular vein Temporal vein Facial vein Common facial vein Others	20 (29.0) 15 (21.7) 13 (18.8) 5 (7.2) 4 (5.8) 4 (5.8) 8 (11.6) 14 (20.3) 14 (20.3) 12 (17.4) 11 (15.9) 6 (8.7) 6 (8.7) 6 (8.7) 36 (52.2) 9 (13.0) 7 (10.1) 4 (5.8) 4 (5.8) 9 (13.0) 35 (50.1) 12 (17.4) 9 (13.0) 7 (10.1) 5 (7.2) 7 (10.1)
Vascular compromise Ischaemia Congestion Reoperation Flap necrosis Total Partial Intraoral flap Sensor detached Extraoral flap Sensor detached	5 (7.2) 4 (5.8) 8 (11.6) 5 (7.2) 3 (4.3) 36 (52.2) 19 33 (47.8) 7

DIEP: Deep inferior epigastric perforator flap, RAMC: Rectus abdominis myo-cutaneous flap, ALT: Antero-lateral thigh flap

**Table 2:** Summary of measurement (n=69).

Total measurement time (s)	41,588 (3,804-86,401)
Mean average blood flow for all patients (ml/min)	8.96 (1.50-34.9)
Mean minimum blood flow for all patients (ml/min)	3.42 (0-16.8)
Mean maximum blood flow for all patients (ml/min)	26.0 (3.7-66.9)

**Figure. 3:**
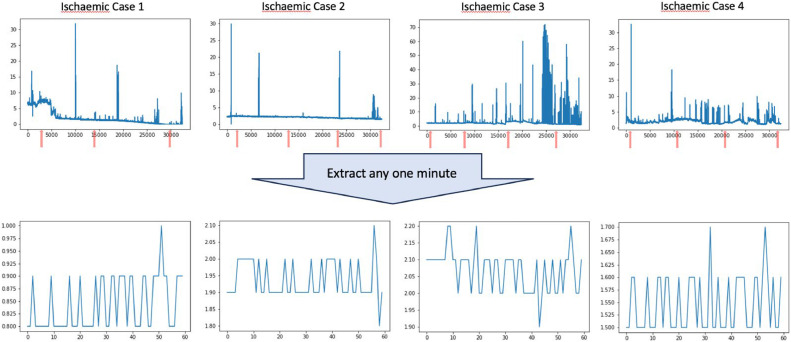
The waveforms for the 4 cases of flap ischaemia for the whole period are shown above and for any 1 min below. The red arrows indicate the timing of the flap assessment by the doctor and last assessment confirmed the diagnosis of ischaemia in all cases.

**Figure. 4:**
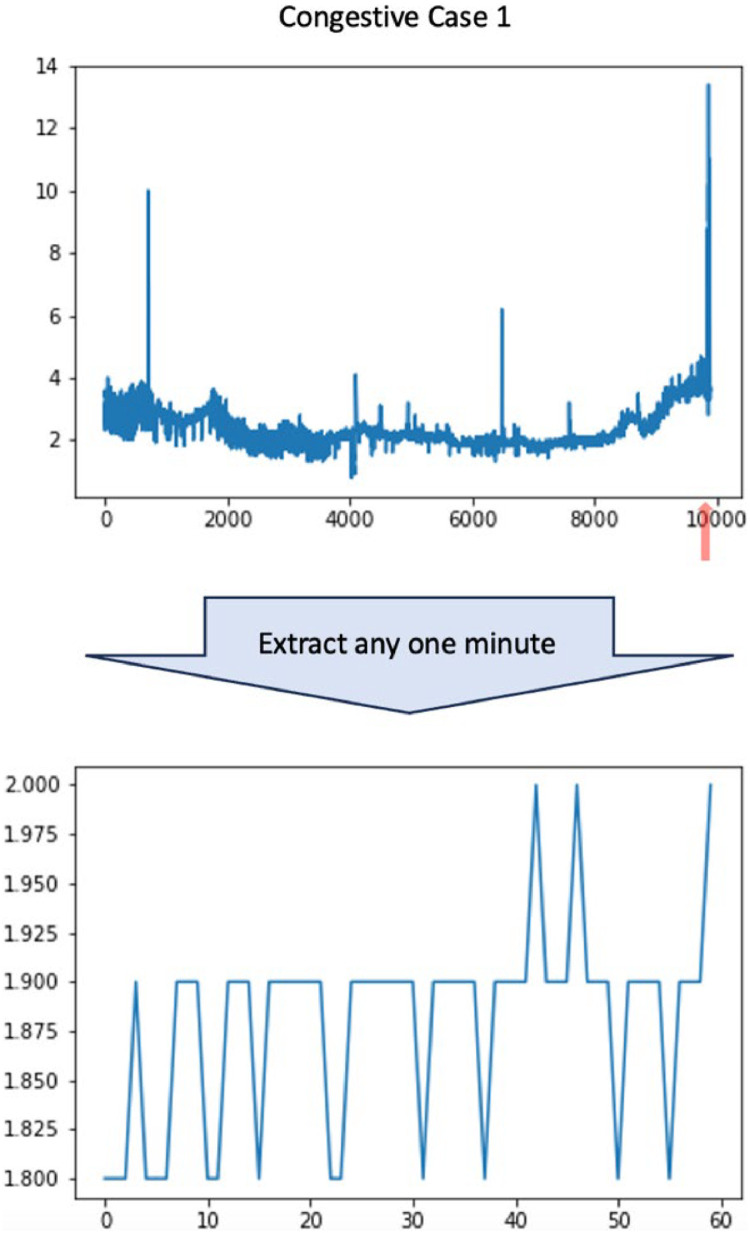
A waveform for flap congestive case.

Arterial ischaemia of the flap was detected in 4 cases and venous congestion in 1 case. The patient background, surgical procedures and outcomes for each case are detailed in Table 3. The waveforms for the ischaemic cases are shown in Figure 3 and for the venous congestive case in Figure 4. Red arrows indicate the times when the flaps were checked by physicians. In all cases, the diagnosis of vascular compromise was confirmed at the final check-up and monitoring was concluded.

**Table 3:** Breakdown of cases with blood flow disorders.

Case Number	Compli- cations	Age (years)	Gender	Indication for Surgery	Method of Free Tissue Transfer	Recipient Vessels	Total Measurement Time (s)	Salvage Procedure	Final Outcome
1	AI	62	M	Exposed hardware after total knee arthroplasty	ALT	Fibular artery (sclerotic due to hypertension), vein	28,075	Re-anastomosis of vessels (failed); flap replacement	Flap replacement
2	AI	46	F	Deformity following surgery and radiotherapy for nasal cavity cancer	RF	Facial artery (revised during procedure), vein	36,652	Patient opted against immediate reconstruction	Decision of delayed reconstruction
3	AI	61	M	Primary tumour excision for lower gingival cancer	Fibula free flap	Superior thyroid artery, internal jugular vein	32,833	Replacement of ischaemic skin paddle with ALT	Flap replacement
4	AI	75	M	Deformity caused by resection of lower gingival cancer; initial reconstruction with a fibula flap and radiotherapy	Fibula free flap	Internal thoracic artery and vein with great saphenous vein grafts	61,496	Replacement of ischaemic skin paddle with LD free flap	Flap replacement
5	VC	80	F	Upper gingival cancer	ALT and PMMC	Superior thyroid artery, external jugular vein	9,897	Re-anastomosis of the vein	Flap survival

AI, Arterial ischaemia, VC, Venous congestion, M, Male, F, Female, ALT, Anterolateral thigh free flap, RF, Radial forearm free flap, PMMC, Pectoralis major musculocutaneous flap, LD, Latissimus dorsi

In 33 of the 69 cases, the flaps were placed extraorally. The sensor was removed or replaced in 26 cases, among which the flap was placed extraoral in 7 cases, significantly less often with an extraoral flap (*p*<0.01, Fisher's exact test).

## Discussion

Despite the established advantages of microvascular free tissue transfer in the reconstruction of complex defects, several studies have underscored the persistent challenges in managing vascular complications. Although free flaps generally exhibit high success rates, approximately 95 % or more,^1^, ^2^, ^3^ the consequence of flap failure due to vascular disturbances cannot be overstated. Such failures compromise the functional and morphological outcomes of the reconstructions, significantly increasing patient morbidity and healthcare costs. In head and neck reconstructions, the risks are especially severe, as flap failures may lead to life-threatening complications or death. Our analysis further highlights the critical importance of timely detection and intervention in enhancing the salvage rates of compromised flaps.

Clinical examination for flap assessment requires considerable clinical expertise and experience, and when performed alone, the survival rate of compromised flaps is in the range of 30-70 %.^24^, ^25^, ^26^ Traditional observations such as checking skin colour, local skin temperature, capillary refilling and bleeding are time-consuming and labour-intensive, thus limiting the ability of experienced microvascular surgeons to perform them frequently.^9^ Historically, most microvascular surgeons have involved resident house staff in flap monitoring protocols and programmes and have relied heavily on residents to ensure timely intervention on compromised flaps.^10^ Meanwhile, the global trend towards optimising resident working hours has forced changes in time-consuming flap assessments, including changes in the frequency of flap monitoring, changes in location (ICU and non-ICU), reliance on ancillary resident staff and has been adapted in several ways, including increased reliance on new technologies for flap checking.^10^

Alternatively, there are negative views on flap assessment, such as clinical monitoring is not cost-effective and should be done selectively,^27^ or that it may disrupt sleep, which is essential for the patients’ recovery from serious illness.^28^

New monitoring technologies enhance care but are not replacements for traditional assessments. Devices such as LDF are crucial adjuncts, particularly in uncertain situations where objective information is needed. Integrating LDF into monitoring protocols can improve detection and management of vascular complications, thereby, potentially increasing the survival rates of compromised flaps. However, the cost of implementing LDF must also be considered. Schönbrunner et al.^29^ noted that Vioptix, a NIRS-based monitoring device, was not cost-effective compared to stand-alone clinical tests. Furthermore, Poder et al.^30^ reported that the Cook-Schwartz Doppler probe would need to be 19 % cheaper to be cost-effective. Kwasnicki et al.^22^ recognised the cost limitations in 13-16 % of the cases involving surveillance devices. However, studies suggest that in breast reconstruction, the cost-effectiveness of clinical monitoring can be justified up to the second post-operative day.^31^ Furthermore, some reports suggest that continuous measurement of vital signs may lead to reduced healthcare costs. In terms of cost, we contacted the companies and found that the NIRS ViOptix is priced at USD 40 700 for a T.Ox dual-channel console and USD 7950 for a five-pack of disposable probes; O2C monitoring monitors cost between EUR 25 000 and EUR 45 000 and probes cost EUR 1850 with a one-year replacement. The LDF device from moor instruments can be installed for £11 000 for the monitor and probe combined. The Pocket LDF® used in this study cost USD 4000 for the monitoring device and USD 670 for the sensor, but running costs were negligible as the sensor is covered with a clear sterile film and can be used repeatedly. The low cost of installation compared to the other devices is a significant advantage in an increasing value-based healthcare system.

LDF is a favoured tool in flap monitoring for its ability to provide non-invasive, immediate qualitative assessments of tissue perfusion. Using the Doppler shift of laser light irradiated onto the flap, LDF deduces the blood flow velocity within the flap, thereby, indirectly assessing the patency of microvascular anastomoses. This assessment is crucial intraoperatively and also during the post-operative phase to aid in decision-making when complications arise^32^. However, the high sensitivity and ease of use of LDF are counterbalanced by its susceptibility to motion and vibration artifacts, which can spuriously elevate flow values^33^. Additionally, the technique's reliability is compromised under conditions of anaemia or haemodilution—common following free tissue transplantation—due to its dependence on erythrocyte density. Maintaining probe contact can be problematic on wet or blood-mixed surfaces, often resulting in probe displacement and falsely low readings.^34^^,^^35^ Another significant limitation is that LDF's penetration depth is only 8 mm^15^, which restricts its applicability, particularly in monitoring buried flaps. Moreover, there is a difficulty in distinguishing between venous and arterial thrombosis from isolated perfusion values.^33^^,^^36^ Furthermore, defining a universal cut-off value that would require prompt intervention is difficult^37^. Ozturk et al. demonstrated that in addition to surgical and clinical factors, such as blood pressure, supplemental oxygen saturation, perforator size and number, flap type and patient demographics, the measurement environment factors including ambient light also had an impact on how well the monitoring device performed.^38^ These factors necessitate a careful interpretation of the LDF data, focusing on trends rather than absolute values to minimise the impact of these limitations.^16^

The result suggests that there was little fluctuation in blood flow in cases of ischaemia and congestion. However, they need to be statistically demonstrated. No distinction was made between ischaemia and congestion, and blood flow did not drop to zero in either case. Clinton et al.^33^ and Yoshino et al.^36^ reported on the difficulty of making this distinction. The issue of interpreting residual LDF signals is well-known and has been described as ‘biological zero’.^39^

Overall, noise due to body movement was prominent. Moellhoff et al.^40^ stated that continuous measurement using an intra-oral probe in patients with free flaps for oral reconstruction was previously impractical from a practicality standpoint. Ooms et al.^34^ employed sutures for intra-oral sensor fixation and reported their usefulness. Future studies on the effects of invasive sensor fixation are warranted.

The result of ischaemia Case 1 also shows that LDF has the potential to provide advance warning of flap deterioration hours before the clinical symptoms manifest. Yoshino et al. reported a sudden drop in blood flow and subsequent necrosis on post-operative days 2 and 3, and Case 1 appeared to have recorded the sudden drop in blood flow. It took 6 h from this drop in blood flow to the decision to reoperate, which was sufficient time for a no reflow phenomenon to occur. It is possible that earlier intervention based on the sensor data would have obviated the need for eventual flap replacement.

Despite its potential benefits, several challenges were encountered with the application of LDF. A significant limitation was the short monitoring duration; typically, monitoring with LDF extends over 48 h. However, due to various constraints in our study, maintaining continuous monitoring for this duration proved difficult. Enhancing the usability of sensors, particularly through the adoption of wireless technology, is crucial for facilitating easier and more reliable long-term monitoring.

Other issues were waveform noises and sensor detachment, particularly noted in sensors placed within the oral cavity. This was a notable concern as the detachment of sensors could lead to inaccurate measurements and compromise monitoring integrity. Therefore, future investigations need to focus on developing non-invasive yet robust adhesive methods that can secure the sensors effectively without causing discomfort or interference.

Additionally, the variability in patient backgrounds and flap heterogeneity could potentially influence the waveform readings and outcomes. Although focusing on waveform trends might reduce the need to consider these variabilities, it is crucial to acknowledge their potential impact on the results.

Furthermore, in this study, LDF was introduced merely as a research tool, and its readings were not used to assess the flaps, which means that we were unable to measure any direct impact on the patient outcomes due to the implementation of this technology. This limits the applicability of our findings in predicting clinical success based on LDF data alone.

The study was conducted in a single facility using a device specific to Japan, which may limit the generalizability of the findings. Therefore, further research involving multiple centres and possibly different devices or techniques is necessary to validate and possibly expand these preliminary findings. This will help in refining the use of LDF technology in free flap monitoring and enhance its reliability and applicability in diverse clinical settings.

In a series of 71 consecutive free flap cases, the Pocket LDF detected 5 cases with flow abnormalities such as ischaemia and congestion. This non-invasive, cost-effective device was suggested to be faster at detecting flow issues than traditional methods. To enhance the predictive accuracy, it is recommended to analyse extensive asymptomatic waveform data using AI and machine learning. Collaborative data collection among the surgeons in a homogeneous setting is essential for reducing data variability and increasing the robustness of findings. Future devices should not only wirelessly transmit anonymised data to the cloud for advanced training but also integrate enhanced sensor technology for more reliable detections. Further research is required to refine the detection of abnormal waveforms and address issues such as sensor detachment to improve the clinical management of flap complications.

## Declaration of competing interest

Hiroki Kodama, MD, Katsuhiro Ishida, MD, PhD, Haruyuki Hirayama, MD, Doruk Orgun, MD, PhD, Dariush Nikkhah, FRCS (plast), James May, BEng, PhD, Panicos A Kyriacou, MSc, PhD, Takeshi Miyawaki, MD, PhD declare that they have no conflict of interest.
